# High-Intensity Focused Ultrasound in Small Renal Masses

**DOI:** 10.1155/2008/809845

**Published:** 2008-12-31

**Authors:** Jose Rubio Briones, Argimiro Collado Serra, Alvaro Gómez-Ferrer Lozano, Juan Casanova Ramón-Borja, Inmaculada Iborra Juan, Eduardo Solsona Narbón

**Affiliations:** Servicio de Urología, Instituto Valenciano de Oncología, C/Beltrán Báguena 8, 46009 Valencia, Spain

## Abstract

High-intensity focused ultrasound (HIFU) competes with radiofrequency and cryotherapy for the treatment of small renal masses as a third option among ablative approaches. As an emerging technique, its possible percutaneous or laparoscopic application, low discomfort to the patient and the absence of complications make this technology attractive for the management of small renal masses. This manuscript will focus on the principles, basic research and clinical applications of HIFU in small renal masses, reviewing the present literature. Therapeutic results are controversial and from an clinical view, HIFU must be considered a technique under investigation at present time. Further research is needed to settle its real indications in the management of small renal masses; maybe technical improvements will certainly facilitate its use in the management of small renal masses in the near future.

## 1. INTRODUCTION

We are facing a rapid increase of incidentally detected small renal masses (SRMs) nowadays [1, 2], prompting us to face many different clinical scenarios and probably minimally invasive ablative techniques will find their role in those unfit patients who are not operable and do not accept a partial nephrectomy or a watchful waiting policy [3] in case of a possible renal cell carcinoma (RCC) diagnosed probably incidentally [4].

High-intensity focused ultrasound (HIFU) induces thermal damage to the targeted tissue without the need of the insertion of a probe into the tissue, thus being the most real “minimally invasive” proposed technique among the ablative treatments for small renal masses (SRMs).

This manuscript will focus on the principles, basic research, and clinical applications of HIFU in small renal masses, reviewing the present literature and analyzing HIFU as a possible treatment for SRM, recognizing no experience in its use for any renal masses by our group.

## 2. MATERIAL AND METHODS

We reviewed PubMed with no limit on time, searching for papers in English or Spanish, using HIFU and renal or HIFU and kidney as key words. We included the literature published on HIFU both in the experimental and clinical settings.

## 3. PRINCIPLES AND TECHNIQUE

The principle of HIFU resembles the one for ultrasound (US), but a higher intensity is used. Ultrasound is progressively absorbed by the tissue and its mechanical energy converted to heat. At a high and focused strategy, the generated heat denatures proteins and produces coagulative necrosis, objectives obtained when temperature reaches 65°C in renal lesions [5]. The induction of thermal necrosis will depend on several factors: the applied power, the US frequency, transducer characteristics (shape, type, size, and number of probes), exposure time, spatial distribution of the field, absorption properties of the tissue, attenuation in the intervening tissue, acoustic reflection and refraction, and finally the perfusion rate in the targeted tissue. Different necrosis rates were shown (volume of ablated tissue per 1 second isonication) for different organs, and for example, the kidney has a lower necrosis rate than the liver [6].

As a technique under development, there are no standard recommendations for its application and this is a point under vast research. Initially, extracorporeal HIFU generators used multiple piezoelectric elements located in a concave disk, generating intensities of >10000 W/cm^2^ which were derived in cavitation lesions, limitating its use in humans [7]. Nowadays, HIFU systems use single transducers, focused by acoustic lenses or by being concave. As the focal lengths are smaller, frequencies of 3-4 MHz can be achieved, producing smaller but better defined lesions. Modern HIFU devices obtain focal depths of 10–16 cm; the focal zones are cigar shaped and the volume ablated depends on power intensity, duration of application, and location of pulses. All this equipment is accompanied by US regular probes trying to control the effectiveness of the HIFU application; interestingly, as power intensity increases (5–20 kW/cm^2^), a cavitation phenomenon appears which permits a target point to monitor HIFU effects [8]. An excellent summary on physical principles and devices of HIFU [9] has recently been published.

In the extracorporeal approach, frequencies of 1–1.8 MHz are used in an attempt to increase penetration; in this range, renal thermal lesions were observed in animal models [10, 11]. In the clinical setting, two systems for extracorporeal HIFU have been tested. First, from Storz Medical (Storz; Schaffhausen, Switzerland), we use a 1 MHz piezo element focused at a depth of 100 mm with a parabolic reflector of 10 cm aperture. An integrated 3.5 MHz B-mode US transducer permits inline imaging of the area to treat. The US beam is coupled into the body by a flexible polyurethane cushion filled with degassed water at 16°C, witch permits the variation of the skin-focal spot distance altering its filling [12]. The second HIFU therapeutic system was designed by Chonqing Haifu CO. Ltd (Chonqing, China); it is composed by a patient table, an operating console, and a treatment unit, situated under the table within a basin filled with degassed water to couple with US delivered to the patient, who lies over the water bath. Exchangeable ellipsoidal transducers of 12 or 15 cm diameter are installed in the water bath around a central 3.5 MHz diagnostic transducer. This system permits frequencies of 0.5, 1.2, and 1.5 MHz and focal lengths of 100–160 mm depending on the transducer used. Following the treatment protocol and by exposing the targeted areas up to six times, the authors achieve an estimated site intensity of up to 20000 W/cm^2^, enough to create cavitation and even bubble formation on real-time diagnostic imaging, which authors propose as successful tissue ablation marker [13].

Due to the problems with extracorporeal HIFU applications that we will further comment on, the equipment moved into the laparoscopic field. In porcine models, it was modified with acceptable partial kidney ablation with no damage to surrounding not targeted tissues [14]. In phase I study, this approach was attempted in the human setting, using conventional lap isolation of the SRM through four 12 mm access ports; authors used intraoperative renal power Doppler US with a 10 Hz laparoscopic US probe (BK Medical, Denmark) to locate the SRM. They then changed one of the ports to an 18 mm port (Ethicon; San Angelo, Tx, USA) to introduce the laparoscopic HIFU system (Sonatherm, Misonix Inc., Fiarmigdale, NY, USA), which is composed by a treatment console, an articulated probe arm, a pomp unit, and the laparoscopic probe (covered with a system which permits cooling with gas-free cold water) [15]. HIFU energy is delivered by a truncated spherical shell 4 MHz transducer with a 30 × 13 mm aperture and a 35 mm focal length. One of the best improvements of this approach, compared to the percutaneous one, is that the probe works in direct contact with the SRM and real-time imaging, based on qualitative assess on hyperechoic changes resulting from boiling and cavitation events, permitting direct control of the procedure with the 12 mm transducer aligned confocally with the HIFU transducer. The procedure was calibrated resembling the results obtained in animal models research to ablate tissue at an average rate of 0.6 cm^3^/min at typical power level between 30–38 W [14, 16].

## 4. RESULTS

We found 42 manuscripts using HIFU and renal/kidney as key words. HIFU has been extensively used in other organs, targeted to malignant and nonmalignant tissues: brain, breast, eye, prostate, bladder, uterus, liver, and so forth, showing no increase in cell dissemination [17–20]. An attractive indication for tumour in a solitary testis has been recently published with acceptable results [21].

### 4.1. Pathological assessment

The thermal damage produced by HIFU causes progressive tissue changes depending on the time when the pathological study is done. Immediately after its application in a porcine model, the tissue demonstrated intense congestion, hyperaemia, and alterations of the micropapillaries, and electron microscopy showed alterations of the mitochondria, ribosomes, and lysozymes. At day 2, necrosis starts to be seen within an intense area of hyperaemia and congestion which results in complete necrosis at day 7. Finally, at day 90, a complete fibrosis of the targeted area is observed [22]. On healthy human kidney, haemorrhages were seen in 15 out of 19 cases and microscopically, it was shown that they were caused by fibre ruptures in the wall of small vessels [23]. In papers where SRMs have been excised after HIFU application, “severe thermal tissue damage” has been defined as intravascular disruption of erythrocyte membranes, vacuolisation of tumour and arterial smooth muscle cells, pycnosis and elongation of tumour cell nuclei, rupture of tumour cell membranes, and cell detachment, changes which correspond to complete tissue necrosis if the time elapsed from HIFU application and specimen removal is longer [24]. Negative NADH staining in snap-frozen tissue obtained before tissue fixation with formaldehyde after HIFU treatment also reaffirms irreversible heat damage [15, 24].

### 4.2. Results in the percutaneous approach

Linke et al. were the first to treat a kidney of a rabbit using extracorporeal HIFU [25]. When applied percutaneously in a rabbit model, it was clearly showed that only 2 out of 9 tumours showed well-demarcated effects of ablation [26]. Watkin et al. treated 18-pig kidneys with acute damage detected in 67% [11]. In a canine model, HIFU application with 400 W power and 4-second pulse duration and a calculated site intensity of 1430 W/h obtained coagulative necrosis of variable degree in the targeted area [12]. Recently, the use of microbubbles injected before percutaneous HIFU isonication of goat kidneys showed better necrosis rates than direct HIFU application [13].

In humans, phase II study using the Storz system was conducted by the University of Vienna. Sixteen renal tumours were treated with HIFU, two with curative intent and 14 were subsequently removed. Examination of the specimens showed poor results in terms of therapeutic effect, as necrosis was found only in 9 out of 14 cases, all of which had been exposed to the highest site intensities, and the histologically damaged tissue only composed 15–35% of the targeted tissue [27]. In another phase II study, Häcker et al. treated 19 patients with RCC before nephrectomy, focusing HIFU to healthy renal tissue; after immediate removal of the kidney, they observed variable but limited pathological signs of thermal damage, as for example haemorrhages, just in 15 out of 19 specimens, but these effects could not be correlated to the energy administered and lesion size did never reach the targeted volume [23].

When using the Chongqing system, Wu et al. applied percutaneous HIFU with a palliative intent in 13 advanced RCC, having shown clinical improvement (less pain and disappearance of haematuria) in most of the treated patients, although treatment was considered incomplete in 10 patients [18]. Similar disappointing results were published from UK, where 8 patients were treated with a similar system and only 4 out of 6 kidneys showed radiological evidence of treatment effect on MRI 12 days after HIFU application and just 1 out of 4 removed kidneys showed histological confirmed ablation [17]. This group is currently undergoing a prospective, nonrandomized clinical trial of percutaneous HIFU in the treatment of SRM, looking at histological outcome in resected tumours in one arm and following the ablated tumours with contrast enhanced MRI in the other arm [28].

### 4.3. Results in the laparoscopic approach

In a recently published clinical phase I study, the laparoscopic HIFU approach previously described was applied to 10 patients with solitary renal masses. Two of them had 9 cm tumours and HIFU was applied just as marker lesion before radical laparoscopic nephrectomy; the rest had SRM with a median size of 22 mm and were treated with a “curative intent” applying HIFU to the entire tumour with a margin of 2-3 mm of surrounding parenchyma. Seven of these tumours were operated afterwards by means of a laparoscopic partial nephrectomy and one was left in situ in a patient with high comorbidities. In the SRM subgroup, a median HIFU exposure time of 19 minutes (range 8–42) was used. The first two patients showed, in the subsequent pathological examination, just a 2-3 mm of vital tissue adjacent to where the HIFU probe was approximated with the rest of the tumour with thermal necrosis; the authors explained this phenomenon to an excessive cooling of the probe, and changing this parameter, they did not observe it again in the remaining cases, although a patient showed a 20% central area with no thermal effects, showing complete thermal necrosis in the 4 remaining removed cases (57%). The nonexcised tumour was successfully treated attending to real-time US data, examination of core biopsies showing thermal necrosis, and follow-up CT scans up to 6 months showing no constraint enhancement and shrinking of the lesion [15].

### 4.4. Complications

There have been just two severe complications due to HIFU application in the abdominal cavity in humans: a superior mesenteric artery infarction and a perforation of the terminal ileum, but both were after treatment of recurrent or metastatic colon carcinoma [29]. When focused to kidneys, no serious side effects have been shown [27]; just 2 patients had grade III skin lesions [28], but the most common type of skin toxicity is less than 1cm blister or track at the treatment site [17]. Changes in laboratory tests are also nonsignificant [17, 18].

## 5. DISCUSSION

 Technology has improved the initial problems of the first HIFU intents to treat kidneys with devices derived from piezoelectric lithotripters [22] which could not focus the targeted lesion; the development of a new HIFU source (Storz UTT System, Storz Medical AG, Kreuzlingen, Switzerland) with a smaller (10 cm) diameter for flexible extracorporeal application permitted the authors to focus precisely on the targeted area in an ex vivo scenario with perfused kidneys, adjusting the pulse duration and the power of the generator to the lesion size [30].

One of the major problems with HIFU is that from an extracorporeal application, there are several factors that interfere between the power emitted by the ultrasound probe and the energy arriving to the targeted area: focal length, type, and characteristics of the tissue to be crossed through variable vascularization of the kidney and its mobility as well as the limitation proximity of air (gut) or bone (ribs) because of reverberation, acoustic shadowing, and refraction [31], the last with burning power with potential damage to close organs.

Another drawback of percutaneous HIFU application is the absence of a reliable radiologic method controlling the effects of HIFU in real time. Research is being done to find more fixed devices coupled with respiratory movements trying to save absorption of ultrasound energy from nontargeted tissues like ribs, fat, or muscles; MRI is being more extensively proposed as a guide to the treatment compared with regular ultrasound due to its information regarding temperature changes in the treated tissue within seconds after application [31]. Unfortunately, movement of the kidney also affects the accuracy of MRI thermometry [32]. Mobility has been partly corrected using multichannel focused US systems, trying to combine motion tracking and feedback electronic steering of the HIFU beam [33] and multiprobe systems of small-aperture confocal HIFU transducers that also theoretically permit more flexible targeting [34, 35].

All these reasons could explain the poor results in the clinical setting, mostly when histopathological assessment of thermal necrosis on the targeted tissue has been studied. The limited clinical experience with the extracorporeal approach and its poor results make this approach not suitable to treat renal cancer in humans, and it has to be considered a technique under experimental research [12].

Although percutaneous approach would be the ideal and real “no invasive,” laparoscopic approach facilitates resolutions of many of the problems facing the percutaneous approach. The use of the 18 mm laparoscopic HIFU transducer, applicable to conventional lap armamentarium and controlled by US, as shown by Klingler et al. in phase I study, indicated just for peripheral tumours not larger than 3.5 cm in size [15], opens a window to clinical research with this method as it really does not clamp the kidney or puncture it as other ablative techniques. Although the protocol is under evolution, the authors have shown safe and promising results with at least better thermal necrosis results than those obtained with the percutaneous approach, but it has to be kept in mind that laparoscopy itself is not complication-free as it needs general anaesthesia, pneumoperitoneum, and tumour isolation, so this approach will have to be compared in randomized trials with other nonablative techniques and also with watchful waiting policies in front of SRM in elderly or unfit patients, the subgroup of patients where it makes sense to avoid open or laparoscopic partial nephrectomy for an SRM.

The follow-up of SRM treated with HIFU is generally performed by contrast-enhanced CT and MRI, but other methods such as PET and microbubble contrast-enhanced ultrasound are under evaluation [36]. Microbubbles increased the ablation efficiency and the visibility of tissue destruction attending to the appearance of hyperechoic regions within the targeted tissue [6]. As with the rest of the nonablative techniques, definitive follow-up protocols are missing [37], and the role of the biopsy in contrast-enhanced lesions has to be investigated [38].

One of the advantages of HIFU applications is that treatments could be repeated, but the need to do it under general anaesthesia results in a limitation of this strategy.

Vast research is needed to establish standards of pulse and power levels which ascertain tissue death, as well as the number and types of probes utilized, as in ex vivo porcine experiments, at identical power levels, lesions induced by multiple probes were larger than those induced by single probe [34]. Another nonresolved issue is the final extent of the coagulative thermal-induced necrosis with time. Finally, extracorporeal or laparoscopic approach will have to define their advantages.

Thus, to establish the clinical usefulness of HIFU to treat SRM, long-term follow-up studies are needed taking into account recurrence-free survival data, quality of life parameters, complications and cost analysis, and all these data compared in clinical trials with open or laparoscopic partial nephrectomy as gold standard techniques [39], cryotherapy and radiofrequency as minimally invasive more developed techniques [40], and watchful waiting policy [3] as options to manage small renal masses.

## 6. CONCLUSIONS

HIFU is a promising approach to treat SRM because it is probably the most minimally invasive among the proposed techniques. Nevertheless, the number of treated patients is very small, and its results with the percutaneous approach make it not applicable to the humans with a curative intent. Laparoscopic approach makes it a loose part of its “minimally invasive” principles, but preliminary data show better thermal necrosis results and better US real-time control of the treatment. For the moment, we think that HIFU has to be considered as an investigational technique. Technical improvements could certainly facilitate its use in the management of SRM in the near future.
